# Compound Qiying Granules alleviates diabetic peripheral neuropathy by inhibiting endoplasmic reticulum stress and apoptosis

**DOI:** 10.1186/s10020-023-00698-3

**Published:** 2023-07-18

**Authors:** Yan Hu, Chen Chen, Zhengting Liang, Tao Liu, Xiaoling Hu, Guanying Wang, Jinxia Hu, Xiaolin Xie, Zhiyan Liu

**Affiliations:** 1 Xinjiang Medical University, Urumqi, 830011 Xinjiang China; 2 Traditional Chinese Medicine Hospital Affiliated With Xinjiang Medical University, Urumqi, 830000 Xinjiang China; 3 Guizhou University of Traditional Chinese Medicine, Guiyang, 550025 Guizhou China

**Keywords:** Diabetic peripheral neuropathy, CQYG, ER stress, Apoptosis, Differentially expressed proteins

## Abstract

**Background:**

Diabetic peripheral neuropathy (DPN) is a major complication of diabetes. This study aimed to investigate the therapeutic effects and molecular mechanisms of Compound Qiying Granules (CQYG) for DPN.

**Methods:**

Rats and RSC96 cells of DPN models were established to evaluate the therapeutic effects of CQYG. Then the morphology and apoptotic changes of sciatic nerves were detected. Further, tandem mass tag based quantitative proteomics technology was used to identify differentially expressed proteins (DEPs) and the underlying molecular mechanisms. Protein expression of key signaling pathways was also detected.

**Results:**

CQYG treatment significantly improved blood glucose and oxidative stress levels, and further reduced nerve fiber myelination lesions, denervation, and apoptosis in DPN rats. Further, 2176 DEPs were found in CQYG treated DPN rats. Enrichment analysis showed that protein processing in the endoplasmic reticulum (ER), and apoptosis were all inhibited after CQYG treatment. Next, CQYG treatment reduced inflammatory factor expression, mitochondrial damage, and apoptosis in RSC96 cells which induced by high glucose. Transmission electron microscopy results found that CQYG treatment improved the morphology of nerve myelin, mitochondria, and ER. CQYG treatment decreased ER stress and apoptosis pathway proteins that were highly expressed in DPN models. In addition, we also predicted the potential targets of CQYG in DEPs.

**Conclusions:**

CQYG exerts neuroprotective effects in experimental diabetic neuropathy through anti-ER stress and anti-apoptosis.

**Supplementary Information:**

The online version contains supplementary material available at 10.1186/s10020-023-00698-3.

## Introduction

Diabetes mellitus (DM) is a chronic metabolic disease. According to the International Diabetes foundation, the global prevalence of diabetes was estimated to be 10.5% in 2021 with approximately 536.6 million people and is expected to increase to 12.2% in 2045 (Sun et al. 2022). Meanwhile, the economic burden of diabetes will reach US $2.1 trillion by 2030 worldwide (Bommer et al. 2018). If uncontrolled in the long term, diabetes is associated with the development of microvascular complications, including diabetic nephropathy, diabetic retinopathy, and diabetic peripheral neuropathy (DPN) (So et al. 2022). Among them, DPN is a heterogeneous disease that can manifest with many different symptoms and, as the most common complication of diabetes, is estimated to affect 50% of diabetic patients (Yorek 2022). DPN manifests as a length dependent sensorimotor polyneuropathy leading to systemic structural and functional alterations in neuronal cells affecting the sensorimotor and autonomic parts of the peripheral nervous system (Chiang et al. 2022). DPN may progress to physical injury with high morbidity and mortality (Shao et al. 2022). Despite the significant impact of DPN on quality of life and health care costs, there is no effective treatment for DPN other than strict glucose control (Hossain et al. 2022).

Despite such a high incidence of DPN, the underlying disease mechanisms of DPN remain to be deciphered. Several pathophysiological mechanisms have been implicated in the development of DPN, including mitochondrial dysfunction, oxidative stress, inflammation, immune dysregulation, and vascular dysfunction, (Markova et al. 2022; Zhuang et al. 2022). DM is a disease typically characterized by hyperglycemia, which is an important factor in neuronal damage. Hyperglycemia induced pyruvate accumulation, followed by reactive oxygen species (ROS) production, further leads to endoplasmic reticulum (ER) stress and DNA damage (Callaghan et al. 2020). Long term ER stress activates c-Jun N-terminal kinase (JNK) and p38 MAPK, thereby promoting apoptosis and necrosis (Lindholm et al. 2017). In addition, ER stress engages mitochondria in the process of apoptosis through Ca^2+^ release (Goel et al. 2022). The canonical function of protein kinase R-like endoplasmic reticulum kinase (PERK) as an ER stress sensor transmits apoptotic signals from the endoplasmic reticulum to the mitochondria during ER stress (Kumar and Maity 2021). Therefore, to achieve effective results, interventions that target ER stress based on apoptosis or oxidative stress need to be developed.

Traditional Chinese medicine (TCM) has a long history for the prevention and treatment of chronic complications such as diabetes. In recent years, several studies have shown that TCM has advantages in treating DPN with fewer adverse effects (Zhang et al. 2022a; Ding et al. 2014). Compound Qiying Granules (CQYG) are in-hospital formulations of traditional Chinese Medicine Hospital of Xinjiang Uyghur Autonomous Region, and their pharmaceutical compositions include Astragalus, polygonatum, Salvia miltiorrhiza, cicadidym, and chickpea, which have the effect of reducing the injured fibers of sciatic nerve (Liu et al. 2017). Preclinical studies have shown that acute and long toxicity experiments in CQYG intervened rats all revealed significant toxic side effects (Hu 2011). However, the molecular mechanism of CQYG in the treatment of DPN still requires continuous exploration. This study explored the ameliorative effects of CQYG intervention on DPN associated endoplasmic reticulum stress, oxidative damage on cellular and animal models.

## Materials and methods

### Experimental animals and models

Eight-week-old male Wistar rats weighing 200 ± 20 g were provided and housed by the animal care center of Xinjiang Medical University. Ambient temperature 23 ± 3 °C, relative humidity 40–70%. After two weeks of free access to water and a standard diet, they were randomly divided into two groups: a streptozotocin (STZ)—induced DM group (T2DM, n = 60) and a control group without DM (n = 12). All animal care and experimental protocols were conducted in accordance with Institutional Animal Care and Use Committee (IACUC), the Xinjiang Medical University (IACUC20200930-9). Control rats were treated with a single intraperitoneal injection of an equal volume of citrate buffer.

The model was constructed as previously described (Davidson et al. 2011). In the DM group, rats were continuously fed with a high-fat diet for 8 weeks. After fasting for 14 h, STZ 30 mg/kg was intraperitoneally injected, and on the third day, STZ 30 mg/kg was again injected. The rats with blood glucose ≥ 11.1 mmol/L were regarded as successful model of DM.

After continuing the high-fat diet for 4 weeks on the basis of DM rats, sensory and motor conduction velocities of the lower extremity sciatic nerves of the diabetic rats were measured using a medical key point-4 work station electromyography machine. A successful rat model of DPN was judged by the determination of more than 11% slowing of sensory or motor conduction velocity after successful modeling in DM rats.

### Treatment

DPN model rats were randomly divided into five groups: DPN, DPN + low dose (1.17 g/(kg d) of CQYG, 2 ml/time by gavage, once a day), DPN + medium dose (2.34 g/(kg d) of CQYG, 2 ml/time by gavage, once a day), DPN + high dose (4.68 g/(kg d) of CQYG, 2 ml/time by gavage, once a day), DPN + TMAO (Trimethylamine oxide; 110 mg/(kg d), 2 ml/time by gavage, once a day). TMAO is a chemical chaperone that binds to its receptor PERK and blunts diabetes induced unfolded protein response activation (Lupachyk et al. 2013; Saaoud et al. 2023). All treatment administration began at the 4 weeks after high-fat diet on DM rats. All rats were continuously administered by gavage for 4 weeks, and all rats were given normal chow feeding, free access to food and water during the administration period. The body weight and blood glucose of the rats were monitored.

### Behavioral test

For mechanical pain behavioral test, the testing environment was a Plexiglas cage of 10 × 20 × 20 cm. The rats were allowed to rest in the cage for 10 min before testing by the von Frey Test, which elicits paw withdrawal responses. The reaction time (s) that elicited a paw withdrawal response was recorded to reflect the mechanical pain sensitivity of the rats.

For hot plate test, rats were placed inside a glass bucket surrounded by a hot plate, and the temperature was adjusted to 55–58 °C. The reaction times for observed behavioral changes such as licking or moving the paw, small jumping, or jumping were recorded. To avoid injury of the paws, they were prohibited from staying in the chamber for more than 10 s.

### Sample collection

The rats were anesthetized by intraperitoneal injection of pentobarbital. Whole blood was collected from the abdominal aorta, and serum was isolated. Sciatic nerves on both sides were collected on the lateral femur of rats. The left sciatic nerves were fixed in formaldehyde or glutaraldehyde, and the right sciatic nerves were stored frozen and stored at − 80 °C. Finally, rats were sacrificed by neck amputation.

### HE and luxol fast blue staining

Formaldehyde fixed sciatic nerve samples were embedded in paraffin, and tissues were subsequently cut into 4 μm transverse sections. Followed by deparaffinization and graded rehydration. Sections were mounted with neutral gum after staining with hematoxylin and eosin. On the other hand, deparaffinized sections were hydrated to 95% ethanol and slides were wet with distilled water for 30–60 s. They were then incubated in 0.1% Luxol fast blue (LFB) solution (servicebio, Wuhan, China) at 60 °C overnight. The sections were blocked with neutral gum. Tissue images were taken with a light microscope (Olympus, Waltham, MA).

### TdT-mediated dUTP-biotin nick end-labeling (TUNEL) staining

Apoptosis in rat sciatic nerves was determined using the TUNEL method. The ApopTag fluorescein in situ apoptosis detection kit (Chemicon, CA, USA) was used according to the manufacturer's instructions. The number of apoptotic cells was determined using confocal fluorescence microscopy (Olympus).

### Transmission electron microscopy

Glutaraldehyde fixed rat sciatic nerves were rinsed with PBS buffer. Dehydration was performed after fixation in 1% osmium tetroxide for 1 h, and tissues were washed with propylene oxide. The tissues were then embedded in epoxy resin. Tissue cut into 60 nm transverse sections using an ultramicrotome. Staining was performed with uranyl acetate and lead citrate. Subsequently examined with a transmission electron microscope (Hitachi, Tokyo, Japan).

### Biochemical indexes detection

Following the instructions of the kits (Nanjing Jiancheng, Nanjing, China), the content of Glu, MDA, SOD, CAT, GSH, and NO in rat serum samples was detected.

### Tandem mass tags (TMT) labeling

Proteins of sciatic nerves of 3 controls, 3 DPN, and 3 DPN + medium dose rats were extracted using SDT (4% SDS, 100 mM Tris–HCl, 1 mM DTT, pH7.6) buffer and quantified using BCA kit. Protein samples were described by trypsin then the resulting peptides were collected. Finally, 100 μg peptide mixture of each sample was labeled using TMT reagent according to the manufacturer’s instructions (Thermo Scientific, IL, US). Samples were randomly distributed between 10-plex label sets, along with 3 each sample per set. Labeled peptides were fractionated by High pH Reversed-Phase Peptide Fractionation Kit (Thermo Scientific).

### LC–MS/MS analysis

LC–MS/MS analysis was performed on a Q Exactive mass spectrometer (Thermo Scientific). The peptides were loaded onto a reverse phase trap column connected to the C18-reversed phase analytical column in 0.1% Formic acid and separated with a gradient of buffer (84% acetonitrile and 0.1% Formic acid). The mass spectrometer was operated in positive ion mode. All tandem mass spectra were generated using the higher-energy collision dissociation (HCD) approach. The MS/MS data were obtained at a resolving power setting of 70,000 at m/z 200, and isolation width was 2 m/z. The MS raw data for each sample were searched using the MASCOT engine (Matrix Science, London, UK; version 2.2) embedded into Proteome Discoverer 1.4 software for identification and quantitation analysis.

### Bioinformatic analysis

Differentially expressed proteins (DEPs) between DPN and controls, or DPN and DPN + medium dose were identified using a *P* < 0.05. gene set enrichment analysis (GSEA) was performed using GSEA software. The intersection of two groups of DEPs was used to construct the co-expression network using weighted gene co-expression network analysis (WGCNA). Enrichment of KEGG pathways were performed using R package “clusterprofiler”. Gene set variation analysis (GSVA) was used to evaluate the activated or inhibited signaling pathways in the DPN groups. Traditional Chinese Medicine Systems Pharmacology (TCMSP) database was used to predict target genes of CQYG.

### Cell culture and treatment

RSC96 rat Schwann cell line was obtained from Procell (Wuhan, China). Cell culture conditions were DMEM (high glucose) + 10% fetal bovine serum (FBS) + 1% penicillin and streptomycin at 37 °C with 5% CO_2_.

The cell proliferation for glucose intervention was detected using CCK-8 kit (TransGen, Beijing, China) following the manufacturer’s instructions. The OD value was gathered at 450 nm. High glucose model was established by intervening RSC96 cells with 150 mM glucose for 24 h, as shown in Table S1. Cells were randomly divided into six groups: RSC96 (control), high glucose, high glucose + low dose (0.01 mg/ml of CQYG pretreated for 24 h), high glucose + medium dose (0.05 mg/ml of CQYG pretreated for 24 h), high glucose + high dose (0.1 mg/ml of CQYG pretreated for 24 h), high glucose + TMAO (5 mM of CQYG pretreated for 24 h).

### ELISA assay

The levels of IL-6, IL-1β, and TNF-a were detected in the RSC96 cell culture supernatants according to the kit instructions (MULTI sciences, Hangzhou, China).

### Flow cytometry assay

Cellular ROS levels were measured using Dihydroethidium (DHE) (5 μM; Biosharp, Hefei, China). RSC96 cells were incubated with DHE for 30 min in the dark, it reacts with superoxide anion to produce red immunofluorescence. For mitochondrial membrane potential detection, RSC96 cells were incubated with JC-1 staining solution (5 μM; Biosharp) for 30 min. Capture images through fluorescence microscopy. ImageJ software 1.38 (NIH, MD, USA) is used to analyze relative fluorescence intensity within cells. In addition, the apoptosis was detected using flow cytometry with Annexin V PE/7AAD kit (BD Biosciences, San Jose, CA).

### Western blot

Tissue samples or RSC96 cells were lysed in RIPA lysis buffer containing protease and phosphatase inhibitor cocktails. Total protein concentration was determined by BCA kit (TransGen, Beijing, China). Equal amounts of protein were separated on SDS-PAGE gels and transferred to polyvinylidene difluoride membranes. After blocking with nonfat dry milk for 2 h at room temperature, the membranes were incubated with primary antibodies (β-actin from mouse (100166-MM10), 1:1000, SinoBiological, Beijing, China; GRP78 from rabbit (3183S), 1:1000, CST, MA, USA; PERK from mouse (3192S), 1:800, CST; p-PERK from rabbit (3179S), 1:600, CST; ATF4 from rabbit (11815S), 1:800, CST; Bax from rabbit (2772T), 1:800, CST; IGF-1 from rabbit (ab223567), 1:1000, Abcam, MA, USA; and CHOP from mouse (2895S), 1:800, CST) overnight at 4 °C. The membranes were subsequently incubated with horseradish peroxidase (HRP)—labeled secondary antibodies (Goat anti-rabbit IgG (ab205719), Goat anti-mouse IgG (ab205718); 1:5000, Abcam) for 2 h at room temperature. Bands were visualized using an enhanced chemiluminescence Kit and an image system (Gel, BioRad, USA). β-actin served as a loading control to normalize the data. The relative expression level of p-PERK was shown as p-PERK/total PERK.

### Statistical analysis

Bioinformatic analysis were performed using R 3.6.1. All data were expressed as mean ± standard deviation. GraphPad Prism 9.3.0 (GraphPad Software, CA, US) was used for data statistics and histogram plots. One-way ANOVA was used for comparison between groups. *P* < 0.05 was considered statistically different.

## Results

### CQYG can improve DPN

**Fig. 1:**
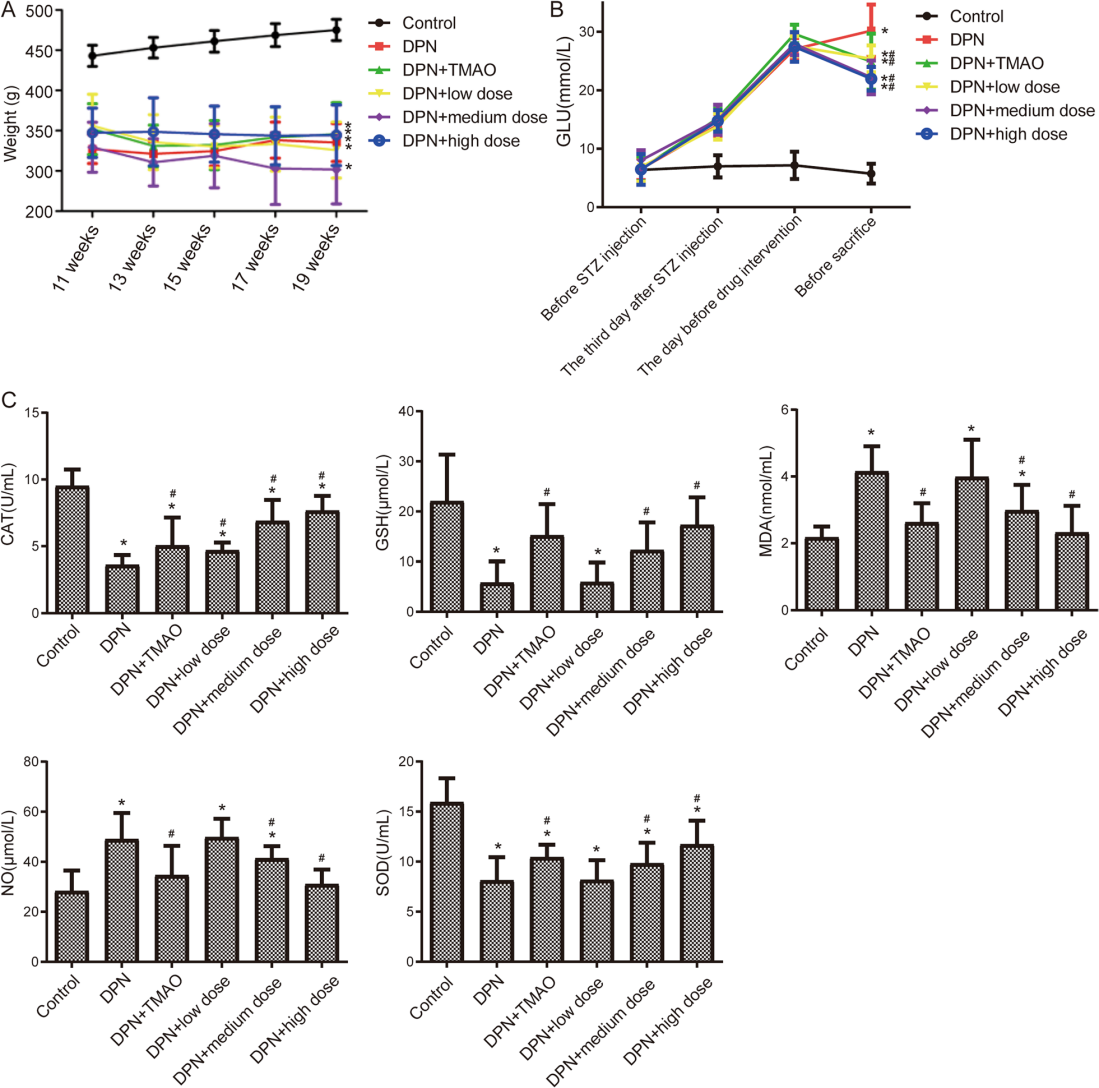
HYPERLINK "sps:id::fig1||locator::gr1||MediaObject::0" CQYG improves DPN. **A** Body weight of each group rats checked for every 2 weeks. **B** Blood glucose of each group rats. **C** MDA, SOD, CAT, GSH, and NO contents in serum of each group rats. *P < 0.05 vs control group; ^#^*P* < 0.05 vs DPN group

The body weight of DPN rats was lower than that of healthy controls, whereas high dose of CQYG and TMAO treated DPN rats slightly higher than that of the DPN rats (Fig. 1A). Detection of blood glucose levels revealed that the blood glucose of DPN rats was significantly higher than that of the control rats, and the blood glucose of CQYG and TMAO treated DPN rats were all significantly lower than that of DPN rats (Fig. 1B). In addition, CQYG and TMAO treated DPN rats significantly reduced the thresholds to thermal stimuli (Table 1) and mechanical stimuli (Table 2) to elicit paw withdrawal responses. Subsequently, we detected oxidative stress in rat serum (Fig. 1C). It was found that the levels of MDA, and NO were significantly increased in DPN rats compared with the controls, whereas they were significantly decreased after the treatment with medium and high doses of CQYG and TMAO. The levels of CAT, GSH, and SOD were significantly lower in DPN rats than that in controls, whereas they were significantly increased after treatment with medium and high doses of CQYG and TMAO.

**Table 1:** Reaction time (s) analysis of hot plate experiment in each group of animals ($\overline{x} \pm s$)

Groups	Four weeks after STZ injection	Four weeks after drug intervention
Control	3.302 ± 0.167	3.183 ± 0.449
DPN	3.245 ± 0.169	3.773 ± 0.522*
DPN + TMAO	3.194 ± 0.153	3.705 ± 0.322*
DPN + low dose	3.253 ± 0.156	4.805 ± 0.659*#
DPN + medium dose	3.285 ± 0.173	3.860 ± 0.328*
DPN + high dose	3.206 ± 0.174	3.660 ± 0.452*

*P < 0.05 vs control group; ^#^*P* < 0.05 vs DPN group

**Table 2:** Analysis of reaction time (s) for the mechanical pain test in each group of animals ($\overline{x} \pm s$)

Groups	Four weeks after STZ injection	Four weeks after drug intervention
Control	3.058 ± 0.268	3.092 ± 0.250
DPN	3.400 ± 0.227*	4.317 ± 0.366*
DPN + TMAO	3.200 ± 0.169	4.413 ± 0.837*
DPN + low dose	3.200 ± 0.131	4.517 ± 0.483*
DPN + medium dose	3.300 ± 0.151*	4.000 ± 0.460*
DPN + high dose	3.313 ± 0.173*	4.000 ± 0.256*

*P < 0.05 vs control group

### CQYG changes DPN pathology

**Fig. 2:**
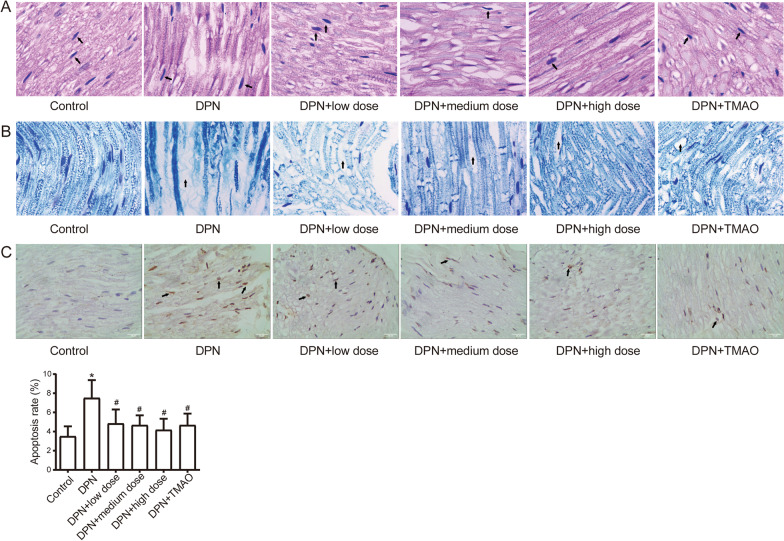
Pathological changes in sciatic nerve tissue. **A** The pathological conditions of sciatic nerve tissues were detected by HE staining. Mononuclear cell infiltration indicated by dark color the arrow pointed. **B** Nerve fiber myelin structure of sciatic nerve tissue was detected by LFB staining. Neuronal demyelination indicated by dark color the arrow pointed. **C** Sciatic nerve tissue apoptosis was detected by TUNEL staining. The apoptosis cells were indicated by dark color the arrow pointed. Bars = 25 μm. *P < 0.05 vs control group; # *P* < 0.05 vs DPN group

The rat sciatic nerves were subjected to HE and LFB staining to observe pathological changes. The results of HE staining showed that axonal degeneration, demyelination, and thickening of the nerve membrane as well as the degree of endoneurial proliferation in the myelinated nerve fibers were significantly improved in DPN rats under the influence of CQYG (Fig. 2A). The results of LFB staining showed that the degree of nerve fiber myelination lesion and loss were both alleviated in rats after CQYG treatment compared with DPN rats (Fig. 2B). In addition, the TUNEL assay results suggested that CQYG treatment significantly decreased the apoptosis rate (Fig. 2C). The above results suggest that CQYG at medium doses has different degrees and aspects of neuroprotective effects on DPN rats.

**Fig. 3:**
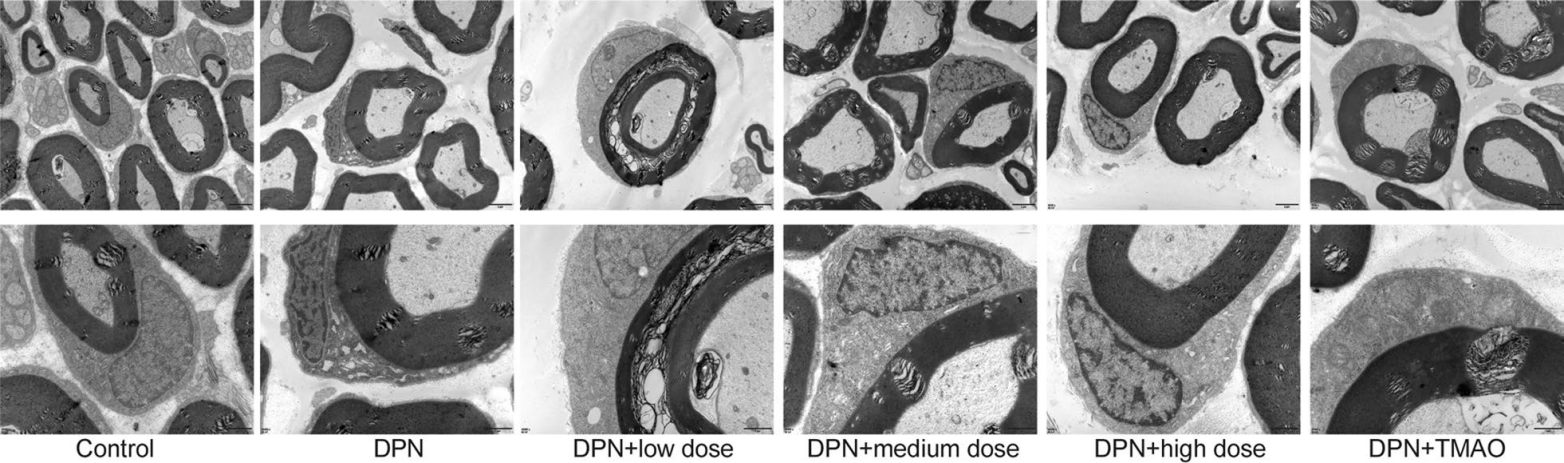
Transmission electron microscopy of rat sciatic nerve. **A** Normal control. **B** Diabetic peripheral neuropathy. **C** Low dose of CQYG treated diabetic peripheral neuropathy. **D** Medium dose of CQYG treated diabetic peripheral neuropathy. **E** High dose of CQYG treated diabetic peripheral neuropathy. **F** TMAO treated diabetic peripheral neuropathy. Up panel: scale bars = 2 μm, down panel: scale bars = 1 μm

To obtain ultrastructural validation of the neuroprotective effects of CQYG, we further utilized scanning transmission electron microscopy to detect ultrastructural changes in rat sciatic nerves (Fig. 3). Cross sections of normal rat sciatic nerves exhibited uniform and dense myelination with intact structure. The myelin sheaths of sciatic nerves of DPN rats were significantly disrupted. Some degree of lamellar breakage, separation of myelin sheath, axonal shrinkage, ridge dissolution and vacuolization of mitochondria, expansion and degranulation of the ER can be seen. The histopathological morphology was improved in DPN + CQYG and DPN + TMAO groups compared with the DPN group.

### Protein expression altered by CQYG in DPN

**Fig. 4:**
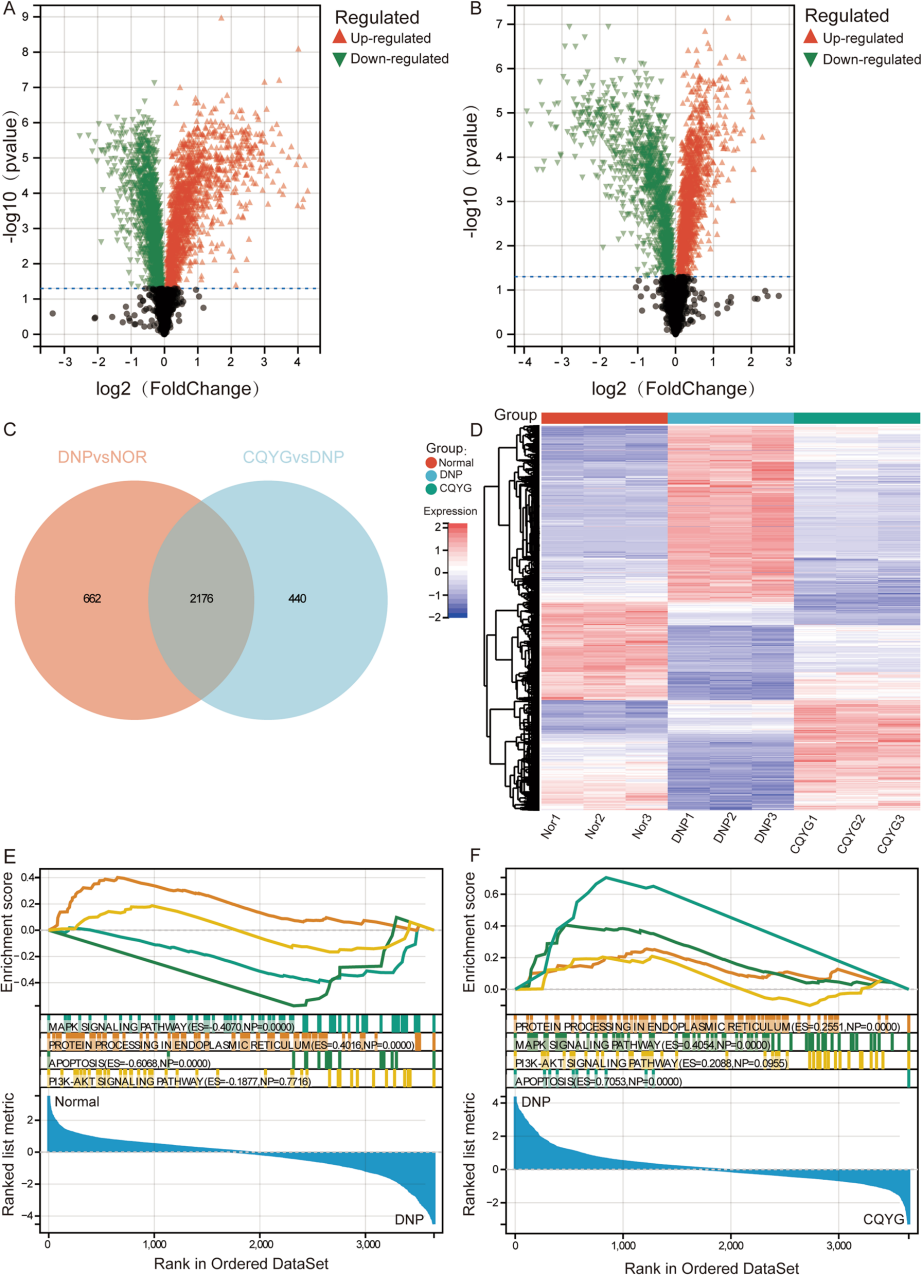
Differential analysis between DPN and normal controls or CQYG treated and DPN group. **A** Volcano plot of differentially expressed proteins between DPN and normal controls. Red is upregulation and green is downregulation. **B** Volcano plot of differentially expressed proteins between CQYG treated and DPN group. Red is upregulation and green is downregulation. **C** Intersection analysis of two groups of differentially expressed proteins to obtained the intersected proteins. **D** Heatmap of intersected proteins in normal control, DPN, and CQYG treated DPN groups. Red is upregulation and blue is downregulation. **E**, **F** GSEA results in DPN **E** and in CQYG treated DPN (**F**). *ES* enrichment score

We next used a proteomics approach of TMT labelling to identify alterations in protein expression caused by DPN and CQYG treatment. Differential analysis results showed that 2838 DEPs were identified between DPN and normal controls (Fig. 4A), 2616 DEPs were identified between the CQYG treated and DPN rats (Fig. 4B). Through intersection analysis, we obtained 2176 intersected DEPs (Fig. 4C). Intersected DEPs expression levels in the three groups of rats found that CQYG treatment improved most of the aberrantly expressed proteins in DPN to the normal direction (Fig. 4D). Furthermore, we found that protein processing in ER, apoptosis, and MAPK signaling pathway were significantly enriched in the DPN group (Fig. 4E) and CQYG treatment group (Fig. 4F) by GSEA.

**Fig. 5:**
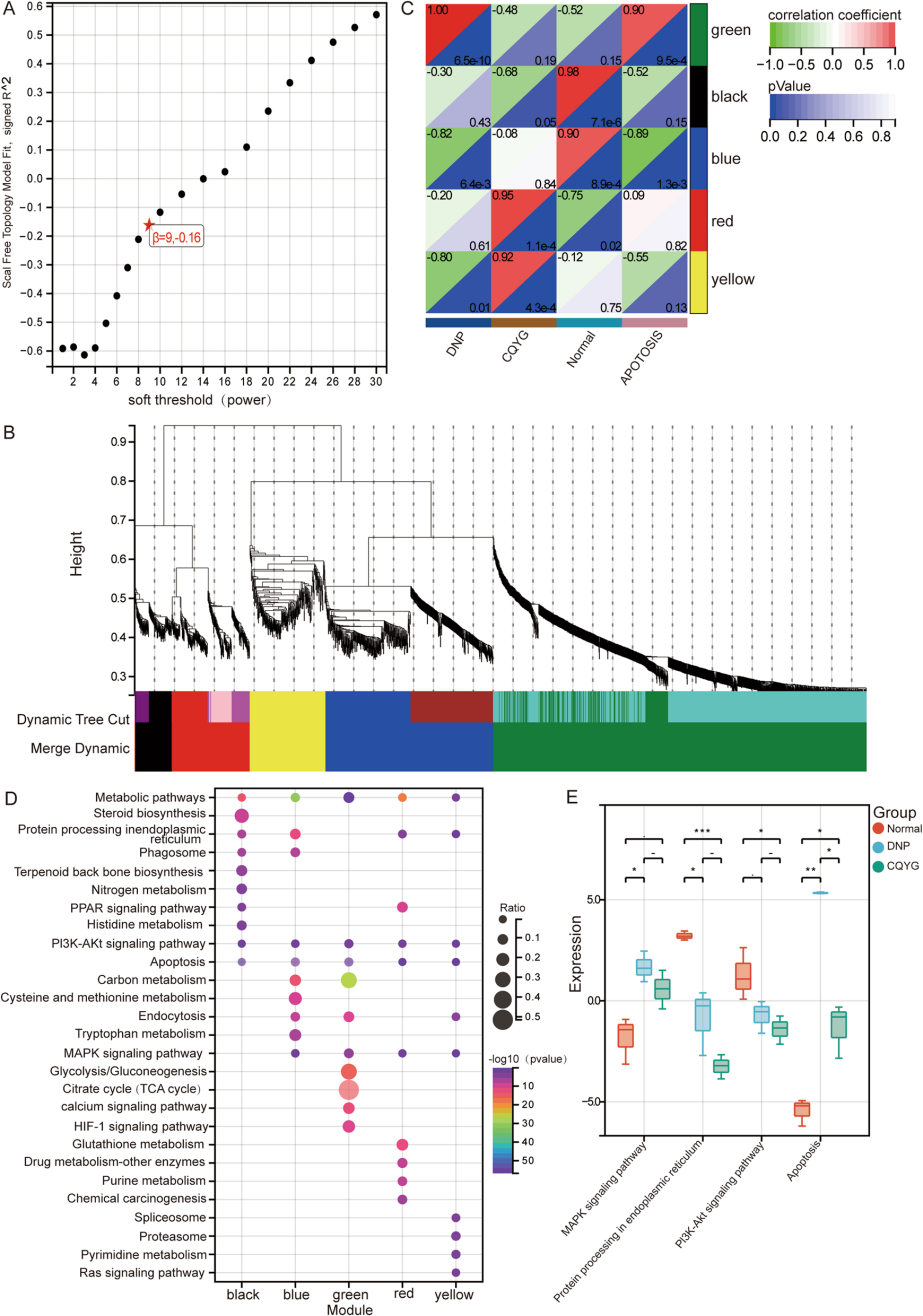
Co-expression network and enrichment analysis of intersected proteins. **A** Selection of soft thresholding powers in WGCNA. **B** Clustering dendrograms of modules for intersected proteins. **C** Correlation between modules and clinical information. **D** KEGG signaling pathways of module genes significantly involved in. **E** GSVA results of significant KEGG pathways in normal controls, DPN, and CQYG treated DPN groups. *P < 0.05, **P < 0.01, ***P < 0.001

In addition, we performed a co-expression network analysis of the intersected proteins using WGCNA. By selecting the soft-thresholding power as 9, 5 co-expression modules were constructed (Fig. 5A, B). Correlation results showed that the green module had the greatest positive correlation between DPN and apoptosis, and negative correlation between CQYG treatment and normal control group (Fig. 5C). Enrichment analysis identified module genes significantly enriched in metabolic pathways, protein processing in ER, PI3K-Akt signaling pathway, apoptosis, and MAPK signaling pathways (Fig. 5D). We performed GSVA to compare with the DPN group, and found that the protein processing in ER, PI3K-Akt signaling pathway, apoptosis, and MAPK signaling pathways were all inhibited after CQYG treatment (Fig. 5E). The above results suggested that CQYG treatment restored the aberrantly expressed proteins in DPN rats and inhibited multiple aberrantly activated signaling pathways.

### Effect of CQYG on mitochondrial damage and apoptosis

**Fig. 6:**
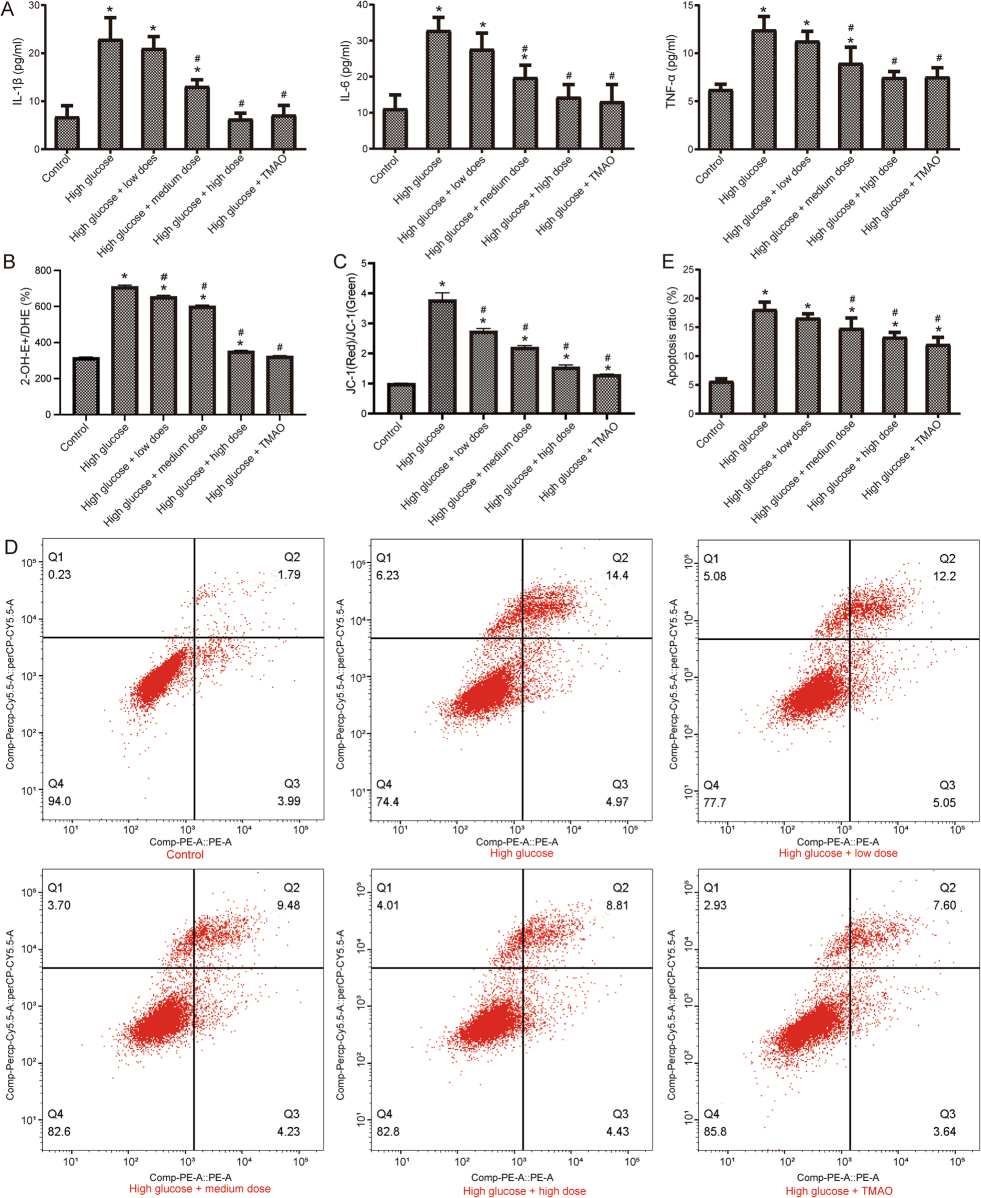
CQYG attenuates neuroinflammation in high glucose injured Schwann cells. **A** ELISA detection of IL-6, IL-1β, and TNF-α levels in RSC96 cells treated with high glucose, CQYG and TMAO. **B** Intracellular ROS levels (Ratios of 2-OH-E + /DHE) in RSC96 cells treated with high glucose, CQYG and TMAO. **C** JC-1 monomer levels (Ratios of red/green) in RSC96 cells treated with high glucose, CQYG and TMAO. **D** Apoptosis detection by flow cytometry in RSC96 cells treated with high glucose, CQYG and TMAO. **E** Apoptosis rate of RSC96 cells. *P < 0.05 vs control group; ^#^*P* < 0.05 vs high glucose group

Next, we used Schwann cells to construct a high glucose model to further explore the effects of CQYG on oxidative damage and apoptosis. Detection levels of inflammatory factors we found that the levels of IL-6, IL-1β, and TNF-α in the RSC96 cells after high glucose induction were significantly higher than those in the control group before induction, while after CQYG treatment, the levels of inflammatory factors in high glucose model were significantly decreased in a dose-dependent manner (Fig. 6A). In addition, we further examined the antioxidant capacity to evaluate high glucose induced oxidative stress and the antioxidant stress effect of CQYG. As presented in Fig. 6B, ROS levels assessed with DHE in high glucose stimulation was elevated compared to controls, and CQYG treatment reduced ROS production. The results of mitochondrial membrane potential detection also found that the JC-1 was also upregulated in high glucose model and downregulated when CQYG treatment (Fig. 6C). For apoptosis detection, CQYG treatment significantly reduced the level of high glucose induced apoptosis (Fig. 6D, E). In conclusion, CQYG attenuated mitochondrial damage and apoptosis in Schwann cells associated with high glucose induced injury.

### CQYG affects ER stress

**Fig. 7:**
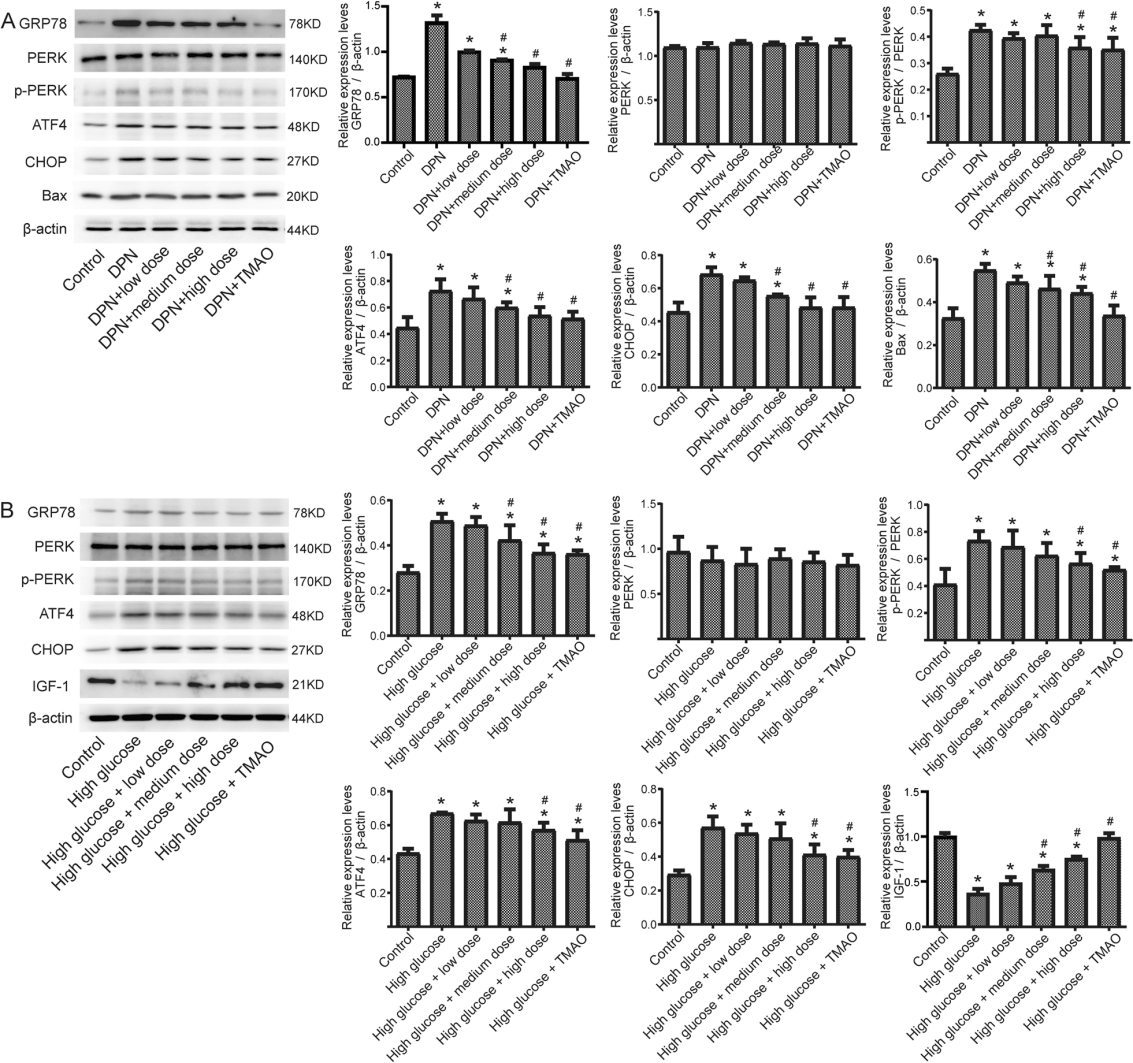
Protein expression in PERK signaling pathway and apoptosis. **A** Important proteins in PERK signaling pathway and apoptosis were detected by Western-blot in different groups of rats. *P < 0.05 vs control group; # *P* < 0.05 vs DPN group. **B** Important proteins in signaling pathway were detected by Western-blot in different groups of RSC96 cells. *P < 0.05 vs control group; ^#^*P* < 0.05 vs high glucose group

To identify the effects of drugs on ER stress and apoptosis, we examined the expression of proteins in pathways using Western-blot. In DPN rats, important proteins in PERK signaling pathway and apoptosis were upregulated than that in controls, and downregulated in DPN + CQYG and DPN + TMAO groups (Fig. 7A). Similarly, CQYG treatment also reduced the level of PERK pathway activation and apoptosis induced by high glucose in the RSC96 cells, and increased IGF-1 expression (Fig. 7B). CQYG may prevent the ultrastructural damage of injured sciatic nerve induced by diabetes and had protective effects on the ER and mitochondria, as well as induced apoptosis.

### CQYG targets in ER stress pathway

**Fig. 8:**
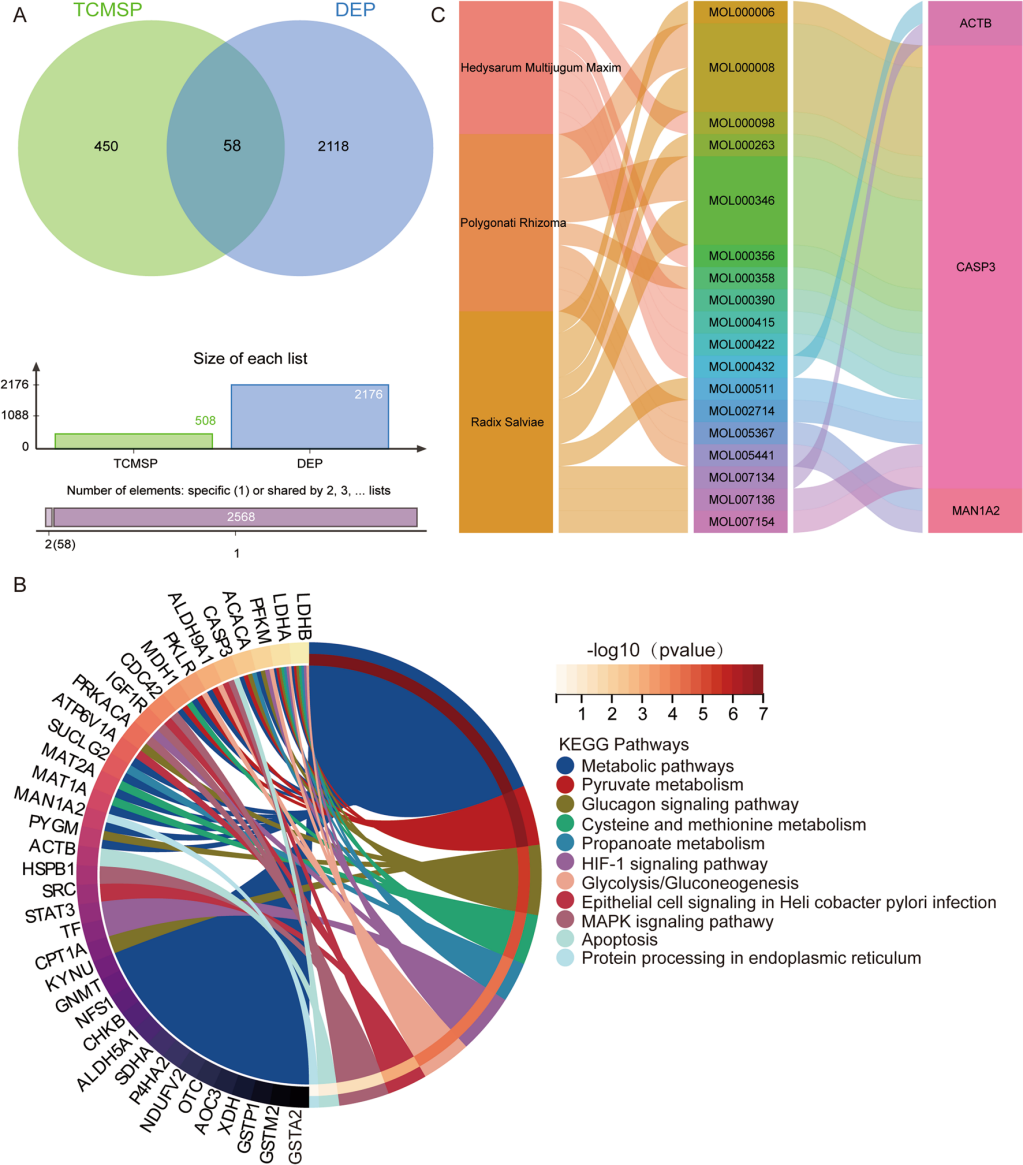
CQYG target prediction. **A** Intersection between CQYG targets in TCMSP database and differentially expressed proteins. **B** KEGG pathways of intersected CQYG targets enriched. **C** Network of connections among compounds, molecules, and differentially expressed proteins

Further, to explore the targets of CQYG in the ER stress and apoptosis, we performed network pharmacology prediction analysis. Using the TCMSP database, we identified 508 target proteins of the herbal components of CQYG, of which 58 proteins were among the DEPs (Fig. 8A). The enrichment results of the 58 proteins showed that actb, Caspase-3, and man1a2 were involved in the endoplasmic reticulum stress and apoptosis pathways (Fig. 8B). Figure 8C demonstrates the network of contacts between herbal components, molecule IDS, and target proteins.

## Discussion

Since the etiology and pathogenesis of DPN remain unclear, there is no specific treatment in the clinic. Finding the best treatment for DPN is a challenging task for physicians. As a major branch of TCM, herbal medicines are widely used to treat diseases. In recent years, TCM has played an increasingly important role in the treatment of complex pathogenesis and symptoms such as DPN (Mai et al. 2021). Effectively controlling early hyperglycemia in DPN can regenerate dysfunctional nerve fibers (Zhang et al. 2022b). Here, we established DPN model rats to further explore that the treatment of CQYG improved the morphology of nerve fiber myelin as well as the level of oxidative stress. Second, protein expression changes in the sciatic nerves of DPN rats after CQYG treatment and is associated with the ER, and apoptosis. It was subsequently further identified in Schwann cells that CQYG treatment caused ER stress, altered expression of apoptosis related proteins. These results suggest that CQYG has a protective effect on the morphology and function of nerve fibers, mitochondria and endoplasmic reticulum caused by DPN.

The results of the present study showed a significant decrease in blood glucose in CQYG treated DPN rats. Whereas CQYG treatment did not improve the overall health index of DPN rats due to > 20% body weight loss in DPN rats. CQYG treatment also produced a protective effect on neurological function in DPN rats, as evidenced by long-term changes in sensitivity to mechanical, cold, and thermal stimuli. Previous studies confirmed that the superoxide dismutase (SOD) content was significantly increased, both malondialdehyde (MDA) and 8-hydroxydeoxyguanosine (8-OHdG) contents were significantly decreased, and nerve conduction velocity was increased in DPN patients after CQYG treatment (Ding et al. 2022). Oxidative damage indicators were similarly significantly improved in DPN rats treated with CQYG. Diabetes induces high levels of lipid peroxidation products, dyslipidemia, and accumulation of toxic metabolites, all of which are associated with oxidative stress (Pang et al. 2020). These will further aggravate mitochondrial damage, leading to overproduction of ROS.

In addition, CQYG treatment markedly reduces the apoptotic index of spinal neurons in the dorsal horn of DPN rats (Abudushalamu et al. 2020). Oxidative stress damages the peripheral nervous system, such as Schwann cells, myelinated axons, and sensory neurons, among others (Chandrasekaran et al. 2019; Fernyhough 2015). ROS can lead to oxidative damage in dorsal root ganglion neurons and is considered a major mechanism in the pathogenesis of DPN (Han et al. 2023). Insufficient mitochondrial energy production can lose the ability to transport axons, further contributing to axonal injury (Rumora et al. 2018). CQYG treatment also improved NO levels in the DPN model. NO causes increased local blood flow and tissue swelling, affecting neuroinflammation and sensitivity in nerve injury (Rosenberger et al. 2020). The results of this study suggest that CQYG has a significant therapeutic effect on the morphological changes of sciatic nerve in DPN rats. Sustained hyperglycemia is thought to induce neuroinflammation and neuronal damage because glycosylation of myelin proteins leads to infiltration of monocytes, neutrophils, and macrophages that further damage the myelin sheath and increase nerve excitability, thus leading to edema and neuroinflammation (King 2001; Shi et al. 2013). Importantly, CQYG treatment promoted the anti-apoptotic efficacy in sciatic nerves. Therefore, it is clinically useful for CQYG therapy to target oxidative stress to ameliorate sciatic nerve injury.

On the basis of these results, we then established a cell model of high glucose injury by Schwann cell simulation. Schwann cells, as primary glial cells in the peripheral nervous system, play crucial roles in physiological and pathological conditions by maintaining nerve structure and function and secreting various signaling molecules and neurotrophic factors to support axonal growth and myelination (Wang et al. 2022a). Studies have shown that Schwann cell injury by high glucose induces oxidative stress, neuroinflammation, and mitochondrial dysfunction, and eventually leads to peripheral nerve injury (Xu et al. 2020). We observed that CQYG dose dependently ameliorated the damage and apoptosis of RSC96 by high glucose.

To explore altered protein expression in the sciatic nerves after CQYG treatment, we performed proteomic sequencing experiments. Five co-expression modules of differentially expressed proteins were obtained by WGCNA analysis. Among them, the green module is a key module involved in the pathogenesis of DPN and response to CQYG treatment. Enrichment analysis revealed that protein processing in the ER, and apoptosis were significantly activated in DPN and were significantly inhibited after CQYG treatment. Neurological, mitochondrial, as well as ER damage was significantly ameliorated by CQYG treatment via electron microscopy. The steady-state cytoplasmic calcium concentration increases in sensory neurons from diabetic rodents, and the subsequent depletion of calcium in the sarcoplasmic reticulum generates ER stress, which can accelerate cell death (Verkhratsky and Fernyhough 2014; Sano and Reed 2013). ER stress has been recognized as a key role of DPN, a mechanism that impairs metabolism, transcriptional regulation and gene expression (Lupachyk et al. 2013; Calcutt 2020). Additionally, dysfunctional ER-mitochondria membrane contact sites, which in turn may generate ER stress, also contribute to peripheral neuropathy (Ozturk et al. 2020). DM chronic hyperglycemia exacerbates apoptosis associated with DPN (Chung et al. 2018). Under injury stress, anti-apoptosis is neuroprotective (Kan et al. 2021). ER stress leads to the activation of PERK and downstream signaling events, such as apoptosis (Wang et al. 2022b). Activation of the PERK pathway, which plays a key role in memory and neurodegeneration (Rozpedek-Kaminska et al. 2020). ER stress-induced PERK activation, also via CHOP, plays an important role in apoptosis in DPN (Yang et al. 2017). The expression levels of ER stress and protein related proteins were significantly increased in both rat and cell models and were significantly decreased after CQYG treatment. Consistent with the results of this study, CQYG treated DPN patients had reduced 2-h postprandial glucose and increased insulin-like growth factor-1 (IGF-1) (Zhang and Hu 2010). IGF-1 can promote axonal growth, reduce neuronal apoptosis, and promote Schwann cell proliferation (Song et al. 2019). Therefore, inhibiting ER stress and concomitant apoptosis may be the best strategy to prevent and regress DPN. Whereas the addition of network pharmacology studies predicts relevant insights into the protein network of CQYG action.

However, the present study still had some limitations. First, the sample size of this study is limited, which may affect the application scalability of key results. Although CQYG is a natural plant and relatively safe, it is a non-defined compound, and the side effects and pharmacokinetics of GSPE need to be further investigated. Additionally, CQYG may ameliorate apoptosis in DPN through ER stress, but the specific mechanism by which CQYG is involved in ER stress remains unclear. Screening for novel neuroprotective components from CQYG should be considered in our future studies.

## Conclusions

In conclusion, we provide a unique framework to identify the therapeutic effects and mechanisms of CQYG on DPN from the perspective of both rat model and cell model. The perspective of protein expression changes is further exploited to expand our understanding of molecular mechanisms. The present results suggest that CQYG have ameliorative effects on nerve injury, ER stress, and apoptosis in response to DNP. Our study highlights a new avenue to develop improved treatment options for DNP.

## Supplementary Information

**Additional file 1:** **Table S1.** Survival rate of glucose intervention detected by CCK-8 kit ($\overline{x} \pm s$, n=3)

## Data Availability

The mass spectrometry proteomics data have been deposited to the ProteomeXchange Consortium via the PRIDE partner repository with the dataset submission reference: 1–20221025-1
