# Supplementary material for: Compound Qiying Granules alleviates diabetic peripheral neuropathy by inhibiting endoplasmic reticulum stress and apoptosis
Source: Mol Med. 2023 Jul 18;29:98. doi: 10.1186/s10020-023-00698-3 (PMC10354983; doi:10.1186/s10020-023-00698-3)
Supplement: Supplementary file 1 — Additional file 1: Table S1. Survival rate of glucose intervention detected by CCK-8 kit ($\overline{x} \pm s$x¯±s, n=3) [file 10020_2023_698_MOESM1_ESM.docx]

Supplementary Material

**Table S1.** Survival rate of glucose intervention detected by CCK-8 kit ($\bar{x}\pm s$, n=3)

| Glucose (mM) | Survival rate (%) | | |
| --- | --- | --- | --- |
|  | 24 h | 48 h | 72 h |
| 25 | 100.000±10.244 | 100.000±6.723 | 100.000±4.474 |
| 50 | 96.891±11.939 | 89.897±3.501^*^ | 94.714±4.887 |
| 100 | 90.171±11.284 | 83.248±4.523^*^ | 78.376±5.443^*#^ |
| 150 | 86.259±7.620 | 64.401±6.315^*#&^ | 68.145±4.941^*#&^ |
| 200 | 70.511±22.185^*#^ | 40.081±2.039^*#&^ | 32.776±3.583^*#&^ |
| 250 | 48.947±6.184^*#&^ | 26.230±7.011^*#&^ | 10.539±2.661^*#&^ |

*P<0.05, compared to 25mM; # P<0.05, compared to 50mM; & P<0.05, compared to 100mM.
